# Supplementary material for: Identification and functional analysis of non-coding regulatory small RNA FenSr3 in Bacillus amyloliquefaciens LPB-18
Source: PeerJ. 2023 May 15;11:e15236. doi: 10.7717/peerj.15236 (PMC10194069; doi:10.7717/peerj.15236)
Supplement: Supplemental Information 4 [file peerj-11-15236-s004.zip › KO/CK-vs-T1_map/map04146.html]

KEGG PATHWAY: Peroxisome - Reference pathway


|  |  |
| --- | --- |
| **Peroxisome - Reference pathway** |  |

[
Pathway menu
| Organism menu
| Pathway entry
| Show description
| User data mapping
]

Peroxisomes are essential organelles that play a key role in redox signalling and lipid homeostasis. They contribute to many crucial metabolic processes such as fatty acid oxidation, biosynthesis of ether lipids and free radical detoxification. The biogenesis of peroxisomes starts with the early peroxins PEX3, PEX16 and PEX19 and proceeds via several steps. The import of membrane proteins into peroxisomes needs PEX19 for recognition, targeting and insertion via docking at PEX3. Matrix proteins in the cytosol are recognized by peroxisomal targeting signals (PTS) and transported to the docking complex at the peroxisomal membrane. Peroxisomes' deficiencies lead to severe and often fatal inherited peroxisomal disorders (PD). PDs are usually classified in two groups. The first group is disorders of peroxisome biogenesis which include Zellweger syndrome, and the second group is single peroxisomal enzyme deficiencies.

|  |  |  |
| --- | --- | --- |
| Reference pathway | 184% 150% 122% 100% 82% 67% 55% | 图片下载 |
